# Supplementary material for: Physician exhaustion and work engagement during the COVID-19 pandemic: A longitudinal survey into the role of resources and support interventions
Source: PLoS One. 2023 Feb 1;18(2):e0277489. doi: 10.1371/journal.pone.0277489 (PMC9891506; doi:10.1371/journal.pone.0277489)
Supplement: S1 Appendix — (DOCX) [file pone.0277489.s001.docx]

**Physician exhaustion and work engagement during the COVID-19 pandemic: A longitudinal survey into the role of resources and support interventions**

***Online Supplementary Materials: S1 Appendix***

**S1 Appendix: Path analyses**

Each regression analysis was built on the basis of a series of increasingly complex models, [1] successively introducing the intercept (null model), control variables (control model), within-level predictor variables (main effects model), and hypothesized interaction effects (interaction model). For all models, we tested model improvement by calculating the change in deviance (i.e., change in –2 times the log-likelihood statistic; -2LL statistic) and the change in variance explained in the outcome variables.

The demographic and control variables that were only assessed at Time 1 were added at the between-level and were grand-mean centered. The control variables that were assessed at each wave were added at the within-level and person-mean centered. Additionally, we included time (coded as 0 to 7) as a covariate because the variables that were assessed at each wave may vary over time.[2] The parameters in all models were estimated using maximum likelihood estimation.

**Summary: Change in Variance**

**Intercept-only baseline models:** We compared two intercept-only models with either fixed or random intercepts. These analyses revealed that inclusion of a random intercept (i.e., allowing for between-person variance) resulted in a better fit to the data. Consequently, we included a random intercept in all analyses.

**Control model:** Adding the control variables led to a decrease of the within-individual residual variance from 0.997 to 0.986 and 0.575 to 0.567 for exhaustion and engagement respectively, indicating that the control variables can explain additional 1.1 (1.4) % of the variance in exhaustion (engagement).

**Main effects model:** Adding the predictor variables led to a decrease of the within-individual residual variance from 0.986 to 0.673, and 0.567 to 0.352 for exhaustion and engagement respectively, indicating that the predictor variables can explain an additional 31.4 (37.4) % of the variance in exhaustion (engagement).

**Interaction model:** Adding the interaction terms in the final model led to a decrease of the within-individual residual variance from 0.673 to 0.662, indicating that 1.1% of the variance was explained by the interaction terms.

**References**

1 Raudenbush SW, Bryk AS. Hierarchical linear models: Applications and data analysis methods (Vol. 1). Thousand Oaks, CA: Sage 2002.

2 Kreemers LM, van Hooft EA, van Vianen AE. Dealing with negative job search experiences: The beneficial role of self-compassion for job seekers' affective responses. *J Vocat Behav* 2018;106:165-179.
