# Supplementary material for: Physician exhaustion and work engagement during the COVID-19 pandemic: A longitudinal survey into the role of resources and support interventions
Source: PLoS One. 2023 Feb 1;18(2):e0277489. doi: 10.1371/journal.pone.0277489 (PMC9891506; doi:10.1371/journal.pone.0277489)
Supplement: S2 Appendix — (DOCX) [file pone.0277489.s002.docx]

**Physician exhaustion and work engagement during the COVID-19 pandemic: A longitudinal survey into the role of resources and support interventions**

***Online Supplementary Materials: S2 Appendix***

**S2 Appendix: Associations between potential control variables and exhaustion and work engagement**

On the within-person level, our analyses revealed that anxiety of COVID-19 infection was positively related to exhaustion (*γ* = .068, *SE* = .020, *p* = .001) and negatively related to engagement (*γ* = -.040, *SE* = .020, *p* = .046). Time was positively related to exhaustion (*γ* = .050, *SE* = .021, *p* = .016) but unrelated to engagement, indicating that participants reported higher exhaustion over time. Survey version was unrelated to exhaustion but positively related to engagement (*γ* = .098, *SE* = .021, *p* < .001), indicating stronger engagement in months without (sickness or holiday) leave.

On the between-person level, trait anxiety was positively related to exhaustion (*γ* = .474, *SE* = .045, *p* < .001), and negatively related to engagement (*γ* = -.269, *SE* = .049, *p* < .001). Learning goal orientation was unrelated to exhaustion but positively related to engagement (*γ* = .314, *SE* = .048, *p* < .001). Gender and job position were unrelated to both exhaustion and engagement and accordingly not included in subsequent models.[3] The control model showed significant improvement in model fit over the null model, (∆χ^2^ = 489.706, ∆df = 14, *p* < .001). Adding the control variables led to a decrease of the within-individual residual variance from 0.997 to 0.986 and 0.575 to 0.567 for exhaustion and engagement respectively, indicating that the control variables can explain additional 1.1 (1.4) % of the variance in exhaustion (engagement).

As shown in Table 2 in the main manuscript, anxiety of COVID-19 infection and trait anxiety related positively to exhaustion (*r*_between_ = .38, *r*_between_ = .45, respectively, *p*’s < .001) and negatively to engagement (*r*_between_ = -.33, *r*_between_ = -.30, respectively, *p*’s < .001). Gender related negatively to exhaustion (*r*_between_ = -.14, *p* = .008), indicating more exhaustion among women. Positive correlations between job position, survey version, and learning goal orientation with engagement (*r*_between_ = .14, *r*_between_ = .15, *r*_between_ = .33, respectively, *p*’s < .01) indicate that medical specialists and participants who had not recently been on leave, reported higher engagement. Finally, exhaustion and engagement were negatively correlated (*r*_between_ = -.60, *p* < .001).

**References**

3 Nezlek J. Diary studies in social and personality psychology: An introduction with some recommendations and suggestions. *Soc Psychol Bull* 2020;15:1-19.
