# Supplementary material for: Physician exhaustion and work engagement during the COVID-19 pandemic: A longitudinal survey into the role of resources and support interventions
Source: PLoS One. 2023 Feb 1;18(2):e0277489. doi: 10.1371/journal.pone.0277489 (PMC9891506; doi:10.1371/journal.pone.0277489)
Supplement: S3 Appendix — (DOCX) [file pone.0277489.s003.docx]

**Physician exhaustion and work engagement during the COVID-19 pandemic: A longitudinal survey into the role of resources and support interventions**

***Online Supplementary Materials: S3 Appendix***

**S3 Appendix: Associations between intervention use and key study variables**

Results at the between-person level (see S3 Table in supplement) showed that participants who experienced more job resources such as managerial support, peer support, and positive feedback used more courses/workshops. Participants who reported relatively more workload and positive feedback used more online information or apps. Participants who reported higher managerial support, self-judgement, and exhaustion used more organized individual support. Participants who reported relatively more managerial and peer support were more involved in organized supportive group meetings. Participants who reported relatively more workload, self-judgment, and exhaustion, and lower job control used more professional support.

Results at the within-person level showed that managerial support was related to the use of organized individual peer support, which means that in months in which participants experienced more managerial support, they were more likely to use organized individual peer support. In a similar vein, in months in which participants experienced lower job control, they were more likely to seek online information and to attend an organized group meeting. Also, in months in which participants reported higher self-judgment, they were more likely to use organized individual support and professional support. Finally, in months in which participants felt more exhausted, they were more likely to seek professional support.
