# Supplementary material for: Physician exhaustion and work engagement during the COVID-19 pandemic: A longitudinal survey into the role of resources and support interventions
Source: PLoS One. 2023 Feb 1;18(2):e0277489. doi: 10.1371/journal.pone.0277489 (PMC9891506; doi:10.1371/journal.pone.0277489)
Supplement: S4 Appendix — (DOCX) [file pone.0277489.s004.docx]

**Physician exhaustion and work engagement during the COVID-19 pandemic: A longitudinal survey into the role of resources and support interventions**

***Online Supplementary Materials: S4 Appendix***

**S4 Appendix: Associations between interventions and exhaustion and work engagement – model testing and growth factor estimates**

For all models, the loadings of the slope factors were fixed to 0 for the time 1 observations, which represents the intercepts or starting levels of exhaustion and work engagement. We modeled a linear pattern of change from this intercept on by increasing the loading of the slope factor by 1 for each subsequent time point. The coefficients of the intercepts were fixed to 1, which is the default parameterization in M*plus*. Furthermore, we allowed covariation between residuals at each time point across the two growth processes to avoid correlations exceeding 1 between the slopes of exhaustion and work engagement.

In the first, initial model, we examined the intercepts (i.e., the starting values) and the slopes (i.e., trajectories reflecting change over time) for exhaustion and work engagement in a parallel latent growth model. We allowed covariation between the intercepts and slopes of exhaustion and work engagement, the intercept of exhaustion (work engagement) and the slope of work engagement (exhaustion), and between both intercepts, and both slopes. The model fit was good with CFI = .970, TLI = .968, RMSEA = .046. The slope factor mean for exhaustion was positive but not significant (*estimate* = 0.019, *p* = .088). The slope factor mean for work engagement was negative but not significant (*estimate* = -0.012, *p* = .157). Both results indicate that, on average, there was no increase or decrease in exhaustion or work engagement over time. However, the variance of both slopes was significant (*estimate* = 0.015, *p* < .001, *estimate* = 0.009, *p* < .001, for exhaustion and work engagement, respectively) indicating that there was variability in slopes across individuals. Results showed that the intercepts of exhaustion and work engagement (*r* = -.625, *p* < .001), and the slopes of both constructs (*r* = -.621, *p* < .001) were negatively associated: higher levels in exhaustion were associated with lower levels in work engagement (and vice versa), and increases in the slope of exhaustion over time were associated with decreases in the slope of engagement (and vice versa). The covariances between the intercept and slope of work engagement and the intercept and slope of exhaustion were negative (*r* = -.269, *p* = .002; *r* = -.226, *p* = .021), indicating that higher initial levels in work engagement and exhaustion were associated with smaller slope values.
