# Supplementary material for: Physician exhaustion and work engagement during the COVID-19 pandemic: A longitudinal survey into the role of resources and support interventions
Source: PLoS One. 2023 Feb 1;18(2):e0277489. doi: 10.1371/journal.pone.0277489 (PMC9891506; doi:10.1371/journal.pone.0277489)
Supplement: S1 Table — (DOCX) [file pone.0277489.s005.docx]

| **S1 Table. Means, Standard Deviations, Intra-Class Correlations, and Intercorrelations Among all Study Variables.** | | | | | | | | | | | | | | | | | | | | | |
| --- | --- | --- | --- | --- | --- | --- | --- | --- | --- | --- | --- | --- | --- | --- | --- | --- | --- | --- | --- | --- | --- |
|  | M | SD | ICC | 1. | 2. | 3. | 4. | 5. | 6. | 7. | 8. | 9. | 10. | 11. | 12. | 13. | 14. | 15. | 16. | 17. | 18 |
| 1. Gender:  Female^a^ | 74.1% | - | - | **-** |  |  |  |  |  |  |  |  |  |  |  |  |  |  |  |  |  |
| 2. Age | 38.61 | 10.41 | - | .23*** | **-** |  |  |  |  |  |  |  |  |  |  |  |  |  |  |  |  |
| 3. Job position: Specialist^b^ | 48.7% | - | - | .14** | .75*** | **-** |  |  |  |  |  |  |  |  |  |  |  |  |  |  |  |
| 4. Fulltime^c^ | 41.3% | - | - | .26*** | -.09 | -.14** | - |  |  |  |  |  |  |  |  |  |  |  |  |  |  |
| 5. Learning goal orientation | 5.20 | 0.80 | - | .01 | .06 | .06 | .06 | - |  |  |  |  |  |  |  |  |  |  |  |  |  |
| 6. Trait anxiety | 3.20 | 1.25 | - | -.13** | -.16** | -.20*** | -.05 | -.10* | - |  |  |  |  |  |  |  |  |  |  |  |  |
| 7. Contact COVID-19 patients^d^ | 0.32 | 0.33 | - | .02 | -.10 | -.02 | .02 | -.06 | -.09 | - | .06** | .09*** | .09*** | .05* | -.02 | -.02 | -.02 | -.02 | -.00 | .03 | -.01 |
| 8. Anxiety COVID-19 infection | 2.60 | 0.61 | - | -.01 | .12* | .01 | .02 | -.06 | .23*** | .01 | - | -.04 | .10*** | .02 | .02 | -.01 | -.01 | .06** | -.03 | .08** | -.05* |
| 9. Survey version^e^ | 0.82 | 0.16 | - | .05 | .02 | .06 | .10 | .04 | -.06 | .04 | -.08 | - | -.02 | -.01 | -.00 | .03 | .01 | -.04* | .03 | -.03 | .09*** |
| 10. Workload | 3.68 | 1.07 | .557 | -.09 | .09 | .09 | -.02 | -.00 | .33*** | .09 | .37*** | -.03 | (.84-.87) | -.05* | -.03 | -.33*** | -.14*** | .37*** | -.22*** | .51*** | -.20*** |
| 11. Managerial support | 4.63 | 1.09 | .542 | .02 | -.32*** | -.24*** | .03 | .14** | -.09 | .03 | -.21*** | .07 | -.26*** | (.91-.93) | .28*** | .26*** | 36*** | -.07** | .22*** | -.12*** | .27*** |
| 12. Peer support | 5.04 | 0.85 | .509 | .03 | -.12* | -.05 | .08 | .20*** | -.20*** | .05 | -.13* | .03 | -.17** | .63*** | (.90-.94) | 1.7*** | .35*** | -.06** | .29*** | -.11*** | .30*** |
| 13. Job control | 4.61 | 0.85 | .464 | .15** | .13** | .14** | .07 | .19*** | -.18*** | -.05 | -.21*** | .07 | -.52*** | .35*** | .34*** | (.76-.83) | .28*** | -.12*** | .28*** | -.29*** | .26*** |
| 14. Positive feedback | 4.87 | 0.80 | .505 | .05 | -.01 | .08 | .08 | .30*** | -.24*** | .03 | -.22*** | .06 | -.33*** | .54*** | .63*** | .56*** | (.80-.88) | -.15*** | .51*** | -.26*** | .55*** |
| 15. Self-judgement | 3.34 | 1.24 | .592 | -.16** | -.06 | -.11* | -.10* | -.02 | .59*** | .03 | .24*** | -.12* | .59*** | -.22*** | -.21*** | -.38*** | -.37*** | (.91-.95) | -.27*** | .30*** | -.24*** |
| 16. Psych. capital | 4.97 | 0.69 | .558 | .12* | .12* | .22*** | .06 | .41*** | -.35*** | .03 | -.28*** | .09 | -.37*** | .44*** | .53*** | .59*** | .77*** | -.48*** | (.77-.86) | -.29*** | .47*** |
| 17. Emotional exhaustion | 3.10 | 1.13 | .526 | -.14** | -.06 | -.09 | .01 | -.08 | .45*** | .06 | .38*** | -.07 | .78*** | -.31*** | -.28*** | -.54*** | -.46*** | .67*** | -.54*** | (.91-.94) | -.38*** |
| 18. Work engagement | 4.56 | 0.91 | .564 | .04 | .01 | .14** | .08 | .33*** | -.30*** | .01 | -.33*** | .15** | -.39*** | .40*** | .48*** | .52*** | .75*** | -.42*** | .73*** | -.60*** | (.78-.87) |
|  | | | | | | | | | | | | | | | | | | | | | |
| Between-level correlations (aggregated across the eight measurement occasions) are presented below the diagonal, within-level correlations are presented above the diagonal. Within-level correlations are standardized and were calculated one by one in M*plus* software. 0 = female, 1 = male; ^b^ 0 = resident, 1 = medical specialist; ^c^ 0 = other than fulltime, 1 = fulltime work; ^d^ 0 = no current contact, 1 = current contact with COVID patients; ^e^ 0 = 1-month version, 1 = 7-day version. Cronbach alpha’s are displayed on diagonal.  **p <* .05, ***p <* .01. ****p <* .001. | | | | | | | | | | | | | | | | | | | | | |
