# Supplementary material for: Physician exhaustion and work engagement during the COVID-19 pandemic: A longitudinal survey into the role of resources and support interventions
Source: PLoS One. 2023 Feb 1;18(2):e0277489. doi: 10.1371/journal.pone.0277489 (PMC9891506; doi:10.1371/journal.pone.0277489)
Supplement: S2 Table — (DOCX) [file pone.0277489.s006.docx]

| **S2 Table. Frequencies of Intervention Involvement Across the Study Period.** | | | | | | | | | | |
| --- | --- | --- | --- | --- | --- | --- | --- | --- | --- | --- |
|  | Intervention use for each time point | | | | | | | | | |
| Type of intervention | | Usefulness | T1 | T2 | T3 | T4 | T5 | T6 | T7 | T8 |
| 1. Course/workshop | | 7.53 (1.04) | 136 (36.0%) | 35 (10.5%) | 95 (27.4%) | 92 (28.2%) | 73 (22.7%) | 37 (12.8%) | 56 (20.8%) | 49 (18.6%) |
| 2. Online information/app | | 6.78 (1.51) | 109 (28.8%) | 47 (14.2%) | 53 (15.3%) | 57 (17.5%) | 35 (10.9%) | 26 (9%) | 12 (4.5%) | 13 (4.9%) |
| 3. Organized individual colleague support | | 7.92 (1.25) | 58 (15.3%) | 43 (13.0%) | 36 (10.4%) | 24 (7.3%) | 23 (7.1%) | 16 (5.5%) | 17 (6.3%) | 14 (5.3%) |
| 4. Organized supportive group meeting | | 7.46 (1.14) | 127 (33.6%) | 72 (21.7%) | 73 (21.0%) | 64 (19.6%) | 61 (18.9%) | 37 (12.8%) | 32 (11.9%) | 35 (13.3%) |
| 5. Professional support | | 8.00 (1.31) | 47 (12.5%) | 33 (9.9%) | 39 (11.2%) | 23 (7.1%) | 39 (12.1%) | 23 (8.0%) | 25 (9.3%) | 28 (10.6%) |
| Due to missing data, *N* varies from 378 to 263. % refer to the valid percentage (i.e., excluding missing data). Usefulness scores (scale 1-10; aggregated across measurements) refer to the mean with the standard deviation in brackets. Due to a technical error, participants (*n* = 5) indicated a grade of 0 for intervention type 2. These scores were recoded into the lowest grade, i.e., a grade of 1. | | | | | | | | | | |
