# Supplementary material for: Physician exhaustion and work engagement during the COVID-19 pandemic: A longitudinal survey into the role of resources and support interventions
Source: PLoS One. 2023 Feb 1;18(2):e0277489. doi: 10.1371/journal.pone.0277489 (PMC9891506; doi:10.1371/journal.pone.0277489)
Supplement: S3 Table — (DOCX) [file pone.0277489.s007.docx]

| **S3 Table**. **Intercorrelations Among Key Study Variables and Intervention Use.** | | | | | |
| --- | --- | --- | --- | --- | --- |
| Intervention categories | | | | | |
| Study variables | 1. Course /  Workshop | 2. Online information /  app | 3. Organized individual peer support | 4. Organized group support | 5. Professional support |
| 1. Workload | .02 (-.03) | .19* (.01) | .12 (.02) | .08 (.04) | .15* (.03) |
| 2. Managerial support | .17* (-.02) | .13 (-.03) | .19** (.09***) | .18** (.00) | -.06 (.01) |
| 3. Peer support | .18* (-.00) | .14 (.03) | .07 (.02) | .15* (.03) | -.08 (.03) |
| 4. Job control | .11 (-.03) | .06 (-.05*) | -.09 (.02) | .05 (-.05*) | -.16* (.02) |
| 5. Positive feedback | .22** (-.01) | .17* (-.01) | -.01 (.06**) | .12 (-.02) | -.06 (.00) |
| 6. Self-judgement | -.04 (-.02) | .06 (.02) | .14* (.08***) | .09 (-.01) | .29*** (.06**) |
| 7. PsyCap | .16* (-.011) | .10 (-.01) | -.10 (-.01) | .03 (.01) | -.11 (-.02) |
| 8. Exhaustion | -.05 (-.01) | .06 (.02) | .17** (.04) | .07 (-.01) | .15* (.05*) |
| 9. Work engagement | .14 (.02) | .04 (-.01) | -.02 (-.01) | .00 (.03) | -.09 (-.02) |
| Within-level correlations are presented in brackets. All correlations are standardized and were calculated in M*plus* software. **p <* .05, ***p <* .01. ****p <* .001. PsyCap = psychological capital. Participation in interventions was indicated with 0 = no, 1 = yes.  To investigate the associations of interventions with the key study variables, mean intervention use was calculated by dividing the total number of interventions that participants used during the study (maximum of 8) by the number of surveys they had completed (maximum of 8). Because incomplete surveys would have distorted the calculation of the mean, we excluded these data points in the analyses. As a consequence, the number of observations included in the correlational analysis slightly deviates from the number of observations included in the multilevel analyses. | | | | | |
