# Supplementary material for: Physician exhaustion and work engagement during the COVID-19 pandemic: A longitudinal survey into the role of resources and support interventions
Source: PLoS One. 2023 Feb 1;18(2):e0277489. doi: 10.1371/journal.pone.0277489 (PMC9891506; doi:10.1371/journal.pone.0277489)
Supplement: S4 Table — (DOCX) [file pone.0277489.s008.docx]

| **S4 Table. Results of Growth Modeling for Exhaustion.** | | | | | | | | | | | | | | | | | | | | |
| --- | --- | --- | --- | --- | --- | --- | --- | --- | --- | --- | --- | --- | --- | --- | --- | --- | --- | --- | --- | --- |
|  | **Model 1 exhaustion** | | | | | | **Model 2 exhaustion** | | | | | | | **Model 3 exhaustion** | | | | | | |
| Predictor variable | Intercept | | | Slope | | | Intercept | | | Slope | | | | Intercept | | | | Slope | | |
| **Control variables** | *Est* | *SE* | *p* | *Est* | *SE* | *p* | *Est* | *SE* | *p* | *Est* | *SE* | *p* | *Est* | | *SE* | *p* | *Est* | | *SE* | *p* |
| Learning goal  Orientation | -.044 | .052 | .405 | .027 | .089 | .760 | -.043 | .052 | .417 | .023 | .089 | .794 | -.040 | | .052 | .448 | .012 | | .087 | .893 |
| Trait anxiety | .492 | .050 | .000 | -.250 | .091 | .006 | .486 | .050 | .000 | -.208 | .091 | .023 | .486 | | .050 | .000 | -.197 | | .090 | .028 |
| Anxiety COVID-19 infection | .273 | .053 | .000 | .088 | .091 | .336 | .280 | .053 | .000 | .097 | .091 | .283 | .278 | | .053 | .000 | .104 | | .090 | .250 |
| Age | -.055 | .081 | .500 | .163 | .134 | .223 | -.090 | .079 | .255 | .139 | .130 | .286 | -.090 | | .079 | .257 | .091 | | .129 | .479 |
| Job position | .062 | .080 | .433 | -.143 | .130 | .271 | .063 | .079 | .426 | -.151 | .129 | .242 | .064 | | .079 | .414 | -.112 | | .130 | .390 |
| Contact COVID-19 patients | .092 | .053 | .081 | .033 | .088 | .705 |  |  |  |  |  |  |  | |  |  |  | |  |  |
| Version | .006 | .053 | .906 | -.109 | .091 | .230 |  |  |  |  |  |  |  | |  |  |  | |  |  |
| Gender | -.074 | .056 | .185 | -.090 | .091 | .322 |  |  |  |  |  |  |  | |  |  |  | |  |  |
| Fulltime work | .068 | .055 | .211 | -.029 | .091 | .747 |  |  |  |  |  |  |  | |  |  |  | |  |  |
| **Intervention variables** |  |  |  |  |  |  |  |  |  |  |  |  |  | |  |  |  | |  |  |
| Professional support |  |  |  |  |  |  |  |  |  | -.131 | .084 | .117 |  | |  |  | -.130 | | .082 | .112 |
| Workshop/course |  |  |  |  |  |  |  |  |  |  |  |  |  | |  |  | -.132 | | .102 | .196 |
| Information/app |  |  |  |  |  |  |  |  |  |  |  |  |  | |  |  | .035 | | .105 | .742 |
| Organized individual support |  |  |  |  |  |  |  |  |  |  |  |  |  | |  |  | -.078 | | .098 | .430 |
| Organized group support |  |  |  |  |  |  |  |  |  |  |  |  |  | |  |  | .274 | | .100 | .006 |
| We report the standardized parameter estimates.  *Model 1*: intercept – slope covariation exhaustion: *estimate* = -0.181, *SE* = 0.115, *p* = .116; intercept – slope covariation engagement: *estimate* = -0.241, *SE* = 0.097, *p* = .013; intercept – intercept covariation: *estimate* -0.582, *SE* = 0.06, *p* < .001; slope – slope covariation: *estimate* = -0.643, *SE* = 0.122, *p* < .001; intercept exhaustion – slope engagement covariation: *estimate* = 0.079, *SE* = 0.119, *p* = .505; intercept engagement – slope exhaustion covariation: *estimate* = 0.172, *SE* = 0.103, *p* = .095.  *Model 2*: intercept exhaustion predicting prof. support: *estimate* = 0.104, *SE* = 0.104, *p* = .316; intercept engagement predicting prof. support: *estimate* = -0.031, *SE* = 0.092, *p* = .735; intercept – slope covariation exhaustion: *estimate* = -0.153, *SE* = 0.118, *p* = .193; intercept – slope covariation engagement: *estimate* = -0.235, *SE* = 0.098, *p* = .016; intercept – intercept covariation: *estimate* -0.554, *SE* = 0.060, *p* < .001; slope – slope covariation: *estimate* = -0.600, *SE* = 0.125, *p* < .001;intercept exhaustion – slope engagement covariation: *estimate* = 0.036, *SE* = 0.121, *p* = .768; intercept engagement – slope exhaustion covariation: *estimate* = 0.129, *SE* = 0.104, *p* = .213.  *Model 3*: intercept exhaustion predicting prof. support: *estimate* = 0.113, *SE* = 0.102, *p* = .269; workshop/course: *estimate* = -0.037, *SE* = 0.106, *p* = .723; information/app: *estimate* = -0.001, *SE* = 0.107, *p* = .991; organized individual support: *estimate* = 0.297, *SE* = 0.103, *p* = .004; organized group support: *estimate* = -0.071, *SE* = 0.108, *p* = .509; intercept engagement predicting prof. support: *estimate* = -0.023, *SE* = 0.091, *p* = .803; workshop/course: *estimate* = .139, *SE* = 0.093, *p* = .138; information/app: *estimate* = 0.078, *SE* = 0.095, *p* = .412; organized individual support: *estimate* = 0.250, *SE* = 0.091, *p* = .006; organized group support: *estimate* = 0.002, *SE* = 0.096, *p* = .979; intercept – slope covariation exhaustion: *estimate* = -0.155, *SE* = 0.122, *p* = .204; intercept – slope covariation engagement: *estimate* = -0.229, *SE* = 0.101, *p* = .024; intercept – intercept covariation: *estimate* -0.555, *SE* = 0.060, *p* < .001; slope – slope covariation: *estimate* = -0.628, *SE* = 0.128, *p* < .001; intercept exhaustion – slope engagement covariation: *estimate* = 0.033, *SE* = 0.124, *p* = .790; intercept engagement – slope exhaustion covariation: *estimate* = 0.155, *SE* = 0.107, *p* = .147. | | | | | | | | | | | | | | | | | | | | |
