# Supplementary material for: Physician exhaustion and work engagement during the COVID-19 pandemic: A longitudinal survey into the role of resources and support interventions
Source: PLoS One. 2023 Feb 1;18(2):e0277489. doi: 10.1371/journal.pone.0277489 (PMC9891506; doi:10.1371/journal.pone.0277489)
Supplement: S5 Table — (DOCX) [file pone.0277489.s009.docx]

| **S5 Table. Results of Growth Modeling for Work Engagement.** | | | | | | | | | | | | | | | | | | |
| --- | --- | --- | --- | --- | --- | --- | --- | --- | --- | --- | --- | --- | --- | --- | --- | --- | --- | --- |
|  | **Model 1 engagement** | | | | | | **Model 2 engagement** | | | | | | **Model 3 engagement** | | | | | |
| Predictor variable | Intercept | | | Slope | | | Intercept | | | Slope | | | Intercept | | | Slope | | |
| **Control variables** | *Est* | *SE* | *p* | *Est* | *SE* | *p* | *Est* | *SE* | *p* | *Est* | *SE* | *p* | *Est* | *SE* | *p* | *Est* | *SE* | *p* |
| Learning goal orientation | .357 | .048 | .000 | -.210 | .084 | .012 | .363 | .048 | .000 | -.214 | .083 | .010 | .362 | .048 | .000 | -.209 | .084 | .013 |
| Trait anxiety | -.232 | .053 | .000 | .126 | .087 | .148 | -.247 | .053 | .000 | .111 | .088 | .207 | -.248 | .053 | .000 | .114 | .088 | .192 |
| Anxiety COVID-19 infection | -.193 | .052 | .000 | -.170 | .087 | .050 | -.196 | .052 | .000 | -.173 | .086 | .045 | -.197 | .052 | .000 | -.173 | .087 | .047 |
| Age | -.070 | .079 | .377 | -.304 | .128 | .018 | -.078 | .077 | .316 | -.306 | .124 | .014 | -.077 | .078 | .321 | -.318 | .127 | .012 |
| Job position | .166 | .077 | .032 | .175 | .125 | .162 | .163 | .077 | .034 | .202 | .123 | .101 | .163 | .077 | .034 | .213 | .126 | .092 |
| Contact COVID-19 patients | .029 | .051 | .566 | -.112 | .084 | .179 |  |  |  |  |  |  |  |  |  |  |  |  |
| Version | .096 | .051 | .060 | -.010 | .088 | .910 |  |  |  |  |  |  |  |  |  |  |  |  |
| Gender | .006 | .054 | .908 | -.037 | .088 | .670 |  |  |  |  |  |  |  |  |  |  |  |  |
| Fulltime work | .085 | .053 | .110 | -.068 | .087 | .430 |  |  |  |  |  |  |  |  |  |  |  |  |
| **Intervention variables** |  |  |  |  |  |  |  |  |  |  |  |  |  |  |  |  |  |  |
| Professional support |  |  |  |  |  |  |  |  |  | .171 | .078 | .029 |  |  |  | .168 | .078 | .032 |
| Workshop/course |  |  |  |  |  |  |  |  |  |  |  |  |  |  |  | -.075 | .099 | .449 |
| Information/app |  |  |  |  |  |  |  |  |  |  |  |  |  |  |  | .056 | .100 | .574 |
| Organized individual support |  |  |  |  |  |  |  |  |  |  |  |  |  |  |  | -.002 | .091 | .979 |
| Organized group support |  |  |  |  |  |  |  |  |  |  |  |  |  |  |  | -.001 | .094 | .994 |
| We report the standardized parameter estimates.  *Model 1*: intercept – slope covariation exhaustion: *estimate* = -0.181, *SE* = 0.115, *p* = .116; intercept – slope covariation engagement: *estimate* = -0.241, *SE* = 0.097, *p* = .013; intercept – intercept covariation: *estimate* -0.582, *SE* = 0.06, *p* < .001; slope – slope covariation: *estimate* = -0.643, *SE* = 0.122, *p* < .001; intercept exhaustion – slope engagement covariation: *estimate* = 0.079, *SE* = 0.119, *p* = .505; intercept engagement – slope exhaustion covariation: *estimate* = 0.172, *SE* = 0.103, *p* = .095.  *Model 2* intercept exhaustion predicting prof. support: *estimate* = 0.104, *SE* = 0.104, *p* = .316; intercept engagement predicting prof. support: *estimate* = -0.031, *SE* = 0.092, *p* = .735; intercept – slope covariation exhaustion: *estimate* = -0.153, *SE* = 0.118, *p* = .193; intercept – slope covariation engagement: *estimate* = -0.235, *SE* = 0.098, *p* = .016; intercept – intercept covariation: *estimate* -0.554, *SE* = 0.060, *p* < .001; slope – slope covariation: *estimate* = -0.600, *SE* = 0.125, *p* < .001; intercept exhaustion – slope engagement covariation: *estimate* = 0.036, *SE* = 0.121, *p* = .768; intercept engagement – slope exhaustion covariation: *estimate* = 0.129, *SE* = 0.104, *p* = .213.  *Model 3*: intercept exhaustion predicting prof. support: *estimate* = 0.113, *SE* = 0.102, *p* = .269; workshop/course: *estimate* = -0.037, *SE* = 0.106, *p* = .723; information/app: *estimate* = -0.001, *SE* = 0.107, *p* = .991; organized individual support: *estimate* = 0.297, *SE* = 0.103, *p* = .004; organized group support: *estimate* = -0.071, *SE* = 0.108, *p* = .509; intercept engagement predicting prof. support: *estimate* = -0.023, *SE* = 0.091, *p* = .803; workshop/course: *estimate* = 0.139, *SE* = 0.093, *p* = .138; information/app: *estimate* = 0.078, *SE* = 0.095, *p* = .412; organized individual support: *estimate* = 0.250, *SE* = 0.091, *p* = .006; organized group support: *estimate* = 0.002, *SE* = 0.096, *p* = .979; intercept – slope covariation exhaustion: *estimate* = -0.155, *SE* = 0.122, *p* = .204; intercept – slope covariation engagement: *estimate* = -0.229, *SE* = 0.101, *p* = .024; intercept – intercept covariation: *estimate* -0.555, *SE* = 0.060, *p* < .001; slope – slope covariation: *estimate* = -0.628, *SE* = 0.128, *p* < .001; intercept exhaustion – slope engagement covariation: *estimate* = 0.033, *SE* = 0.124, *p* = .790; intercept engagement – slope exhaustion covariation: *estimate* = 0.155, *SE* = 0.107, *p* = .147. | | | | | | | | | | | | | | | | | | |
