# Supplementary material for: Physician exhaustion and work engagement during the COVID-19 pandemic: A longitudinal survey into the role of resources and support interventions
Source: PLoS One. 2023 Feb 1;18(2):e0277489. doi: 10.1371/journal.pone.0277489 (PMC9891506; doi:10.1371/journal.pone.0277489)
Supplement: Attachment — Submitted filename: Response to Reviewers.docx [file pone.0277489.s010.docx]

**Academic Editor suggestions:**

**#1** The design chosen does not allow for actual effectiveness estimations. However, if considering comparative effectiveness from repeated-measurements observational data, highly we encourage to consider adding propensity scores. Please avoid the use of the section interventions in the methods sections since not facing here a clinical trial or to be describe differently. Same goes for the effectiveness in the results section. Consider including the study design in the title and rephrase it.

***Response***

*We thank the editor for these helpful remarks. We agree that our design does not justify statements on effectiveness. Therefore, and in line with the editor’s suggestion, we have adjusted the headings (page 18, lines 348-349; page 20, lines 378-379) as well as the text in both the methods and the results section (page 12, lines 261-262; page 20, lines 381-382; 392-393; page 21, lines 415-417; page 22, lines 438-440) and now refer to associations between interventions and our key study outcomes rather than effectiveness throughout the manuscript. We also have revised the section ‘interventions’ in the methods section in line with the editor’s remark on clinical trials and have changed this heading into ‘assessment of intervention use’ (page 10, line 228), implying that intervention use is measured rather than manipulated.*

*We thank the editor for the suggestion to use propensity scores and have taken that suggestion seriously. We have come to the following conclusion, however. Propensity scoring has the same function as including covariates, namely providing conditional effect sizes given a set of third variables. Although we acknowledge that there are advantages of propensity scoring - for instance it eliminates the assumptions that relations are linear (as the use of covariates assumes) - it also has serious downsides. Since it is a reduction technique (a set of covariates is being reduced to a single one), potentially important information is being lost.*

*We did, however, include possible confounds in all our analyses, such as demographics, trait anxiety, and goal orientation (see page 14, line 296). Also, there are no theoretical reasons to expect that the findings of our study are flawed by a confounded involvement of participants in the different interventions. We believe that from a substantive point of view, the inclusion of covariates, as we have applied, will provide the reader with results that are better interpretable. For this reason, we judge the use of covariates superior.*

*Finally, as suggested, we have revised the title as to include the study design: ‘Physician exhaustion and work engagement During the COVID-19 pandemic: A longitudinal survey into the role of resources and support interventions’ (page 1, lines 4-6).*

**#2** Pay attention to table 1 suggestion from reviewer. Please consider moving some of the vast statistics table to the supplementary files also. Consider de use of a flow diagram.

***Response***

*We thank the reviewer for these remarks. We have removed table 1 from the methods section and have included it as suggested by reviewer 2 in the results section (page 13). Furthermore, also in line with the suggestions from reviewer 2, we have moved table 2 (i.e., correlation table) and table 4 (i.e., intercorrelations among study variables and intervention use) to the supplementary (S1 Table and S3 Table). Also, as suggested by reviewer 2, we have revised the results section as to simplify it for the reader. Specifically, we have removed the descriptions of explained variance both in the preliminary and main analyses and have moved these to the supplement (page 2-3, lines 38-53). These revisions concern the following sections:*

*Page 14, lines 292-295: ‘Furthermore, we compared two intercept-only models with either fixed or random intercepts. These analyses revealed that inclusion of a random intercept (i.e., allowing for between-person variance) resulted in a better fit to the data. Consequently, we included a random intercept in all analyses.’*

*Page 14, lines 303-306: ‘Adding the control variables led to a decrease of the within-individual residual variance from 0.997 to 0.986 and .575 to .567 for exhaustion and engagement respectively, indicating that the control variables can explain additional 1.1 (1.4) % of the variance in exhaustion (engagement).’*

*Page 17, lines 318-321: Adding the predictor variables led to a decrease of the within-individual residual variance from 0.986 to 0.673, and .567 to .352 for exhaustion and engagement respectively, indicating that the predictor variables can explain an additional 31.4 (37.4) % of the variance in exhaustion (engagement).’*

*Page 18, lines 343-345: ‘Adding the interaction terms in the final model led to a decrease of the within-individual residual variance from .673 to .662, indicating that 1.1% of the variance was explained by the interaction terms.’*

*Finally, we have added a flow chart (Fig 2; page 13, line 282).*

**#3** Ensure to adapt STROBE to the CHERRIES (https://pubmed.ncbi.nlm.nih.gov/15471760/) to have the full view of the questionnaire process.

***Response***

*We thank the editor for this comment. We have adapted the STROBE checklist to the CHERRIES checklist, now displaying the full questionnaire process.*

**Reviewer Comments to the Author**

**Reviewer #1**: This is an original article investigating the role of resources and support Interventions in the development of work exhaustion among physicians during the COVID-19 pandemic. The study presents the results of original psychometric research in an intelligible fashion and is written in standard English. The methodology is explained in detail and is appropriate for this type of research. Statistical analyses are performed to a high technical standard and are described in sufficient detail. There are no ethical issues to be addressed. Conclusions are presented in an appropriate fashion and are supported by the data. My opinion is that this paper should be accepted for publication without further corrections.

***Response***

*We thank the reviewer for the positive evaluation of our manuscript and are very happy to read that this reviewer recommends publication of our manuscript.*

**Reviewer #2**: General comments: The thematic of the research is interesting, and it was well conducted. However, adjustments in the writing of the article must be made before approval.

***Response***

*We thank the reviewer for this positive remark. In response to your comments, we have made several adjustments in the writing. Please, see our responses to each of your comments.*

**#1** Introduction
I felt the lack of a focus on the scenario of COVID-19 as an aggravating factor for the exhaustion of professionals, and make it clearer in the introduction which support interventions will be studied.

***Response***

*We thank the reviewer for this critical remark and agree that it is important to stress the context in which our study was conducted (i.e., the COVID-19 pandemic) as an aggravating factor influencing healthcare professionals’ well-being. We have revised the introduction section of the manuscript as follows:*

*Page 4, lines 91-100: ‘Around the world, healthcare workers -particularly those on the frontline- have been greatly impacted both physically and mentally by the COVID-19 pandemic: staff shortages leading to long working hours, a lack of protective equipment, seeing many patients die, the risk of contracting COVID-19, and worries about colleagues, family, and friends have taken a toll on healthcare workers’ mental health. Indeed, several studies found that healthcare workers’ mental health has worsened during the pandemic, with many reporting symptoms of depression, anxiety, and distress, [14-16] and an increasing risk of physician burnout [17]. In order to combat physician burnout and thus ensure patient safety [18] in times of crisis and beyond, an important first step is to understand which factors can protect physicians’ well-being.’*

*Moreover, we have revised the introduction section as to detail the different type of interventions that are studied (page 6, lines 151-155):*

*‘Here we investigate if different interventions aimed at support and personal development (i.e., a class or workshop, an app or online information, individual peer support, group support, and professional support from a mental health expert or coach) can help to mitigate burnout symptoms and revive engagement. [38-40]’. Additionally, we have moved Table S1 from the supplement to the main manuscript (now Table 1, page 11) as to highlight the different type of interventions we study.*

**#2** Methods
I suggest removing the entire description of the population, including the table, since they are results, from the second paragraph of the methodology.

***Response***

*We thank the reviewer for this suggestion and have moved the description of the study sample, including the table, to the results section (page 12-14, line 272-284).*

Results

**#3** The description of the results is very extensive, and difficult to read. I suggest the authors to be more direct and clear in the results; a detailed description of the statistical issue isn’t necessary.

***Response***

*We agree that the results are extensive and complex. To simplify the results for the reader, we have revised the results section and have moved some of its content to the supplementary.*

*Specifically, we have moved the description of explained variance as a result of model complexity to the supplementary (page 14, lines 303-306; page 17, lines 318-321; page 18, lines 343-345). Moreover, we made some smaller adjustments to the results section as to simplify it for the reader (page 14, lines 292-295; page 21, lines 419-420). We have decided to keep the model fit information and model estimates as these concern vital information of our analyses and are expected to help the reader understand and interpret the study findings. Finally, also in line with the editor’s suggestion, we have moved table 1 and 4 to the supplementary (now S1 and S3 Table in supplement)*

**#4** Focus on how to support interventions would improve this situation, which is the objective of the study.

***Response***

*We thank the reviewer for this remark. We have revised the manuscript as to highlight that understanding the impact of (support) interventions is one of the main goals of our study. To that end, we have moved the S1 Table (summarizing the different interventions, now Table 1) from the supplementary to the main manuscript (which is also in line with the reviewer’s first comment) and have revised the manuscript as following:*

*Page 6, lines 151-155: ‘Here we investigate if different interventions aimed at support and personal development (i.e., a class or workshop, an app or online information, individual peer support, group support, and professional support from a mental health expert or coach) can help to mitigate burnout and revive engagement.’*

*Page 18, lines 351-354: ‘One of the key goals of this study was to understand how participation in support programs relates to physicians’ well-being over time. To that end, we assessed participants’ use of interventions, the extent to which they perceived interventions as useful, and how interventions related to* *exhaustion and work engagement.’*

**#5** Discussion:
• I consider important to cite other studies carried out on the subject and to make comparisons with their results

***Response***

*We agree with the reviewer that it is important to include other relevant studies on the subject. Apart from the studies mentioned in the introduction section (page 6, lines 134-155), we have included other relevant studies in the discussion section and discuss how their findings relate to ours. We have revised the manuscript as follows:*

*Page 24, lines 483-484: ‘This finding is in line with previous intervention studies indicating that interventions are taken up especially in times when resources are low and strain is high [35,73]’*

*Page 25, lines 496-499: ‘This is in line with previous findings indicating that receiving professional support (including goal-setting and action-planning) helped physicians to strengthen personal resources and reduce burnout (76).’*
